# Gel Protein Extraction’s Impact on Conformational Epitopes of Linear Non-Tagged MPT64 Protein

**DOI:** 10.3390/gels9070578

**Published:** 2023-07-14

**Authors:** Sri Agung Fitri Kusuma, Muhammad Fadhlillah, Tina Rostinawati, Intan Timur Maisyarah, Raden Indah Puspita Syafitri, Toto Subroto

**Affiliations:** 1 Department of Biology Pharmacy, Faculty of Pharmacy, Padjadjaran University, Sumedang 45363, Indonesia; t.rostinawati@unpad.ac.id (T.R.); intan.timur@unpad.ac.id (I.T.M.); radenindah16@gmail.com (R.I.P.S.); 2 Study Center of Drugs Dosage Form Development, Faculty of Pharmacy, Padjadjaran University, Sumedang 45363, Indonesia; 3 Department of Chemistry, Faculty of Mathematics and Natural Sciences, Padjadjaran University, Sumedang 45363, Indonesia; putudinata@gmail.com (M.F.); t.subroto@unpad.ac.id (T.S.); 4 Research Center of Molecular Biotechnology and Bioinformatics, Padjadjaran University, Bandung 40132, Indonesia

**Keywords:** MPT64, passive elution, electroelution, non-tagged, intracellular

## Abstract

The production and purification of recombinant proteins are crucial to acquiring pure MPT64 protein. Due to the fact that protein epitopes may undergo conformational changes during purification, this study, therefore, investigated an effective rapid purification method to produce highly intracellular pure MPT64 protein without causing conformational changes in the epitope under denaturing conditions. MPT64 was isolated from *E. coli* and electrophoresed using gel SDS-PAGE. Then, the desired protein bands were excised and purified with two methods: electroelution and passive elution. The isolated protein was identified via peptide mass fingerprinting using MALDI-TOF MS and reacted with IgG anti-MPT64, and the cross-reactivity of the isolated protein with IgY anti-MPT64 was confirmed using Western blot. The results show that both of these methods produced pure MPT64 protein, and the MPT64 protein was confirmed based on the MALDI-TOF MS results. Neither of these two methods resulted in epitope changes in the MPT64 protein so it could react specifically with both antibodies. The yield of MPT64 protein was higher with electroelution (2030 ± 41 µg/mL) than with passive elution (179.5 ± 7.5 µg/mL). Thus, it can be inferred that the electroelution method is a more effective method of purifying MPT64 protein and maintaining its epitope than the passive elution method.

## 1. Introduction

The Mycobacterium tuberculosis protein 64 (MPT64) is an extracellular protein that is specifically released by the *M. tuberculosis* complex and employed as a detectable antigen for tuberculosis diagnosis utilizing a rapid test kit including a particular antibody [1,2,3,4,5]. In the current study, MPT64 was generated in *E. coli* as a recombinant protein that was tag-free for protein purification. It should be emphasized that *E. coli* also manufactures other intracellular proteins in addition to our target protein. The expressed proteins in *E. coli* can comprise up to 50% of the total cellular protein. Hence, purification is more difficult since recombinant proteins are mostly synthesized inside the cells [6]. Moreover, inclusion bodies are frequently formed when recombinant protein molecules aggregate during high-level production in *E. coli* [7,8,9]. Large-scale recovery of bioactive proteins is a significant difficulty due to inclusion body formation in bacterial hosts. It takes a lot of work to extract bioactive proteins from inclusion bodies, and the yields of recombinant proteins are frequently poor [10]. The intended protein is frequently expressed at a high translational rate when high temperatures, high inducer concentrations, and strong promoter systems are used during protein production. As a result, the bacterial protein quality control mechanism becomes completely depleted, and the partly folded and improperly folded protein molecules clump together to form inclusion bodies [11]. Therefore, in order to obtain pure antigens, the secreted MPT64 protein needs to be separated. *E. coli* recombinant protein manufacturing and purification is frequently accompanied by pricy and challenging processes, particularly for medicinal proteins [12]. The production and purification of recombinant proteins are crucial processes, particularly for obtaining pure antigens. A pure protein can be prepared as an antigen using a variety of purifying techniques. Upstream production and downstream purification are two typical distinct processes. The latter is frequently time-consuming and expensive due to the need for expensive chromatography columns (often requiring multiple chromatography steps) or specific affinity resins that use affinity tags like, for example, His, GST, or Strep, which require additional protease treatment and chromatography for tag removal [13]. In this study, the MPT64 protein needed to be obtained as a pure protein, but since it was built without any tags to make purification using column affinity chromatography easier, we thus used sodium dodecyl sulfate–polyacrylamide gel electrophoresis (SDS-PAGE) to isolate the MPT64 protein into a single band on the gel. However, in the purification stage using column affinity chromatography, the target protein is often found to be contaminated with other proteins. Therefore, a simplified procedure with less difficult stages is required to purify the desired protein. The requirement for lengthy chromatographic purification can be reduced if a practical and effective method of retrieving the desired protein molecules is created. Thus, the requirement for tags and several chromatographic stages can be reduced.

The purification of recombinant proteins needs to be carefully planned since it has a substantial influence on protein function and application in addition to being the cheapest stage in MPT64 production. Recombinant protein purification (RPP) and characterization can be difficult, costly, and time-consuming, yet they are necessary to establish the quality of a protein. This means that an effective downstream processing step is crucial for a recombinant protein’s successful use. This includes the purification, verification of the quality, measurement, and storage of proteins. The initial stage in the entire RPP approach should be the creation of a rational protein purification method. Because sequence additions might conflict with a poor purification design, misfolded or heterogeneous protein samples may arise. An ideal antigen must be a highly pure protein with very little (less than 1%) contamination. An in-gel separated protein can be regarded as devoid of large-size contaminants, even if impurities are still present at low concentrations [14]. A specific protein of interest must be removed from a gel’s matrix in order to be obtained from the gel. Therefore, after being extracted from the polyacrylamide gel and identified, the MPT64 protein can be distinguished from other proteins based on its molecular weight. To prevent the chemical alteration of the eluted proteins, a gentle approach to eluting the proteins into a liquid phase is necessary. Elution via passive diffusion and electroelution are frequently used strategies [15,16]. Electroelution is a purification method that prevents contamination by other proteins that could occur during other protein purification methods like chromatography [17,18]. However, the easiest way to release protein molecules from a gel matrix is by adding water (or a buffer) to a portion of the gel that contains the desired protein. This procedure is known as elution via diffusion or passive elution [19,20]. This study investigated an effective rapid purification method to produce highly intracellular pure MPT64 protein without causing conformational changes in the epitope under denaturing conditions.

## 2. Results and Discussion

### 2.1. MPT64 Overproduction

In recent years, there has been tremendous expansion in the variety of products and processes available for the manufacture and purification of recombinant proteins. Recombinant proteins are increasingly being used in industrial settings and academic research, and for medicinal and diagnostic purposes [21,22]. For diagnostic purposes, one of the components of the TB diagnostic kit that we devised was independently made in order to support the national independence of medical devices. In this study, we produced the MPT64 target protein by over-expressing the synthetic MPT64-encoding gene in *E. coli* BL21 (DE3). The most often utilized host microbe in biotechnology is *Escherichia coli*. *E. coli* can be used to produce heterologous proteins because of a number of benefits. Despite these benefits, making recombinant proteins in *E. coli* has certain drawbacks, such as the development of inclusion bodies, ineffective protein translocation, and metabolic cost [21]. Bacterial growth is a complicated process that involves metabolite synthesis (anabolic) and the breakdown (catabolic) of various cell components [22]. The MPT64 protein used in this investigation was produced as a recombinant protein without a tag for protein purification. Thus, the pelB signaling peptide was fused to this MPT64 protein to be released into the medium. In our earlier investigation, the MPT64 protein was translocated across the host cell membrane of *E. coli* by using pelB as a signal peptide [23]. Another study that utilized the same signal peptide and cell host reported the effectiveness of pelB in releasing protein into a medium [24]. Nevertheless, the yield of the MPT64 protein extracellular recovery was still poor in our prior investigation, and a large amount of this protein remained trapped in the cytoplasm and periplasm [23]. In accordance with this, it has been extensively documented that the periplasm is always where the challenge of expressing extracellular proteins in *E. coli* is located [25]. The acquisition of extracellular MPT64 protein may be boosted by enhancing the leaking of certain host cell membranes by engineering the cell host into a leaky phenotype using triton X-100 (0.5% *v*/*v*), which is how we planned to bypass this problem. However, the high level of MPT64 protein would still be confined in the cytoplasm and periplasm [26]. Therefore, to improve the efficiency of antigen uptake in our investigation, we employed intracellular MPT64 protein. But in addition to producing our target protein, *E. coli* also creates other intracellular proteins that are spontaneously spilled into the cytoplasm.

In this investigation, the MPT64-overproducing plasmid pD861-SR:319895 was used to examine the growth behavior of recombinant E. coli BL21 (DE3) cells. Mycobacterium tuberculosis complex (MTBC) secretes a unique protein called MPT64 that sets it apart from other mycobacteria that do not cause tuberculosis (MOTT). Therefore, this protein can be employed as the primary element antigen of a diagnostic method to find M. tuberculosis in a clinical sample.

**Figure 1:**
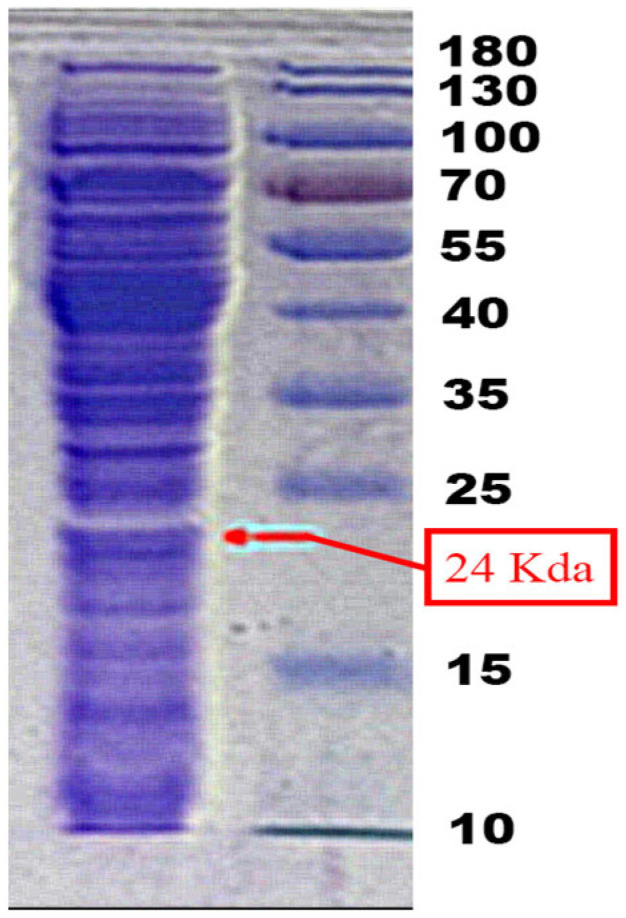
Intracellular MPT64 protein characterization using SDS-PAGE.

In this research, the MPT64 protein was generated as a recombinant protein without a tag for protein purification. The characterization that was carried out on the overproduced result of MPT64 protein recombinant intracellular fractions can be seen in Figure 1. MPT64 protein was obtained at 24 KDA according to the theoretical size of MPT64, and we observed that E. coli also makes other intracellular proteins that are spontaneously discharged into the cytoplasm in addition to expressing our target protein. Therefore, to acquire the pure antigen, the secreted MPT64 protein had to be separated. The most crucial consideration in triggering a particular immune response to an antibody is antigen purity [23,24].

### 2.2. MPT64 Purification

The MPT64 protein used in this study was not designed with His-tag fusion to purify the protein. Recombinant proteins are often created by attaching a tag to the target protein to make it easier to separate the target protein from the host cell’s total protein extract using affinity-based chromatography methods. However, the MPT64 protein utilized in this work was not made with any tag fusion to make it easier to purify. However, the disadvantages of affinity chromatography are highly time-consuming, expensive for scale-up and potent to cause loss of the purified substance [27]. Moreover, as noted in several investigations, nickel-column affinity chromatography protein purification occasionally fails to completely remove impurities in the target protein from other protein bands. This is because His-tag splits using nickel-column affinity chromatography are not 100% efficient. Therefore, we propose that SDS-PAGE can be used as a straightforward approach to generate highly pure MPT64 protein extraction. The MPT64 protein can be isolated from the polyacrylamide gel, distinguished from other proteins based on their molecular weights, and characterized to determine its precise protein identification.

**Figure 2:**
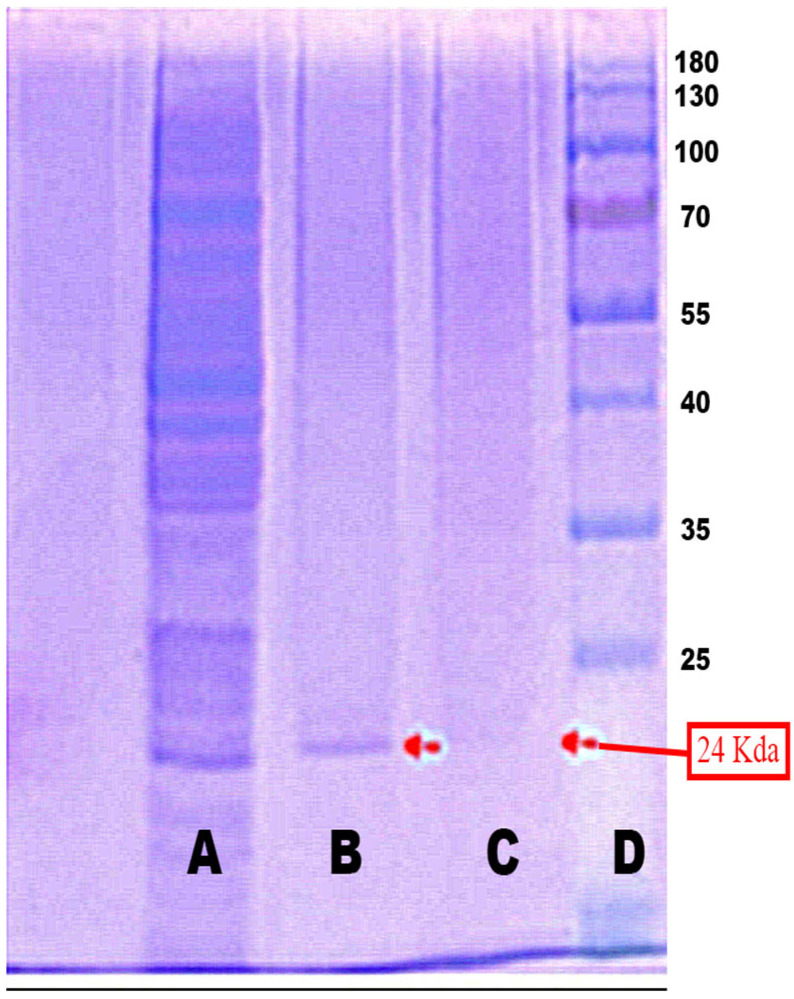
Intracellular MPT64 protein characterization using the electroelution method. (**A**) Intracellular MPT64 protein before purification. (**B**) Electroelution purification with 12 bands. (**C**) Electroelution purification with 9 bands. (**D**) Protein marker.

**Figure 3:**
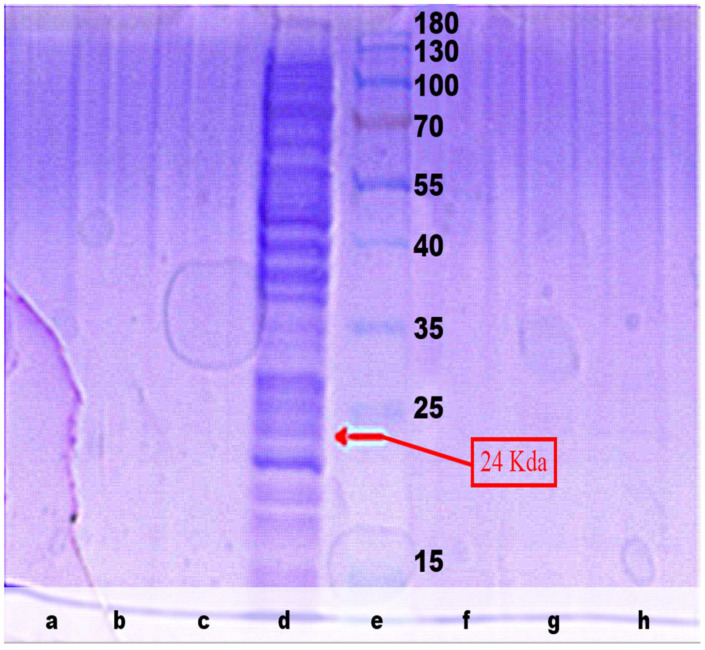
Intracellular MPT64 protein characterization using the passive elution method (**a**) Purification with 6 bands for 96 h. (**b**) Purification with 9 bands for 96 h. (**c**) Purification with 12 bands for 96 h. (**d**) Intracellular MPT64 protein before purification. (**e**) Protein marker. (**f**) Purification with 12 bands for 72 h. (**g**) Purification with 9 bands for 72 h. (**h**) Purification with 6 bands for 72 h.

In this study, the goal of producing purified MPT64 protein was to use it as the main component in antigen-based diagnostic kits for tuberculosis in the future. Hence, antigen purity was the most important factor to consider for generating a specific immune reaction [28,29]. However, as can be seen in Figure 1, the intracellular protein extract contained a variety of proteins with different sizes that could be the same. Therefore, extra or other intracellular protein removal steps are usually required to obtain pure MPT64 protein. Electroelution and passive elution are the procedures that are most often employed. The protein can be extracted by eluting it from the gel phase into the liquid phase. This extraction technique has the benefit of not subjecting the eluted target protein to denaturation or chemical modification [15,30]. The presence of contaminant-free MPT64 protein can be monitored throughout the purification process via electrophoresis, as presented in Figure 2 and Figure 3. If there is a single band at the correct size (24 Kda), this is a good indication of pure MPT64 protein that is free of protein contamination. But due to the concentration limit being too low, the MPT64 protein purity extracted using the passive elution technique could not be adequately detected using SDS-PAGE electrophoresis. This is in line with the data in Table 1, indicating that the electroelution approach, which uses a total of 12 protein band isolates, yielded a substantially higher protein content than the passive elution method, which uses a variety of protein band counts with a longer time period of extraction. This emphatically demonstrates that the electroelution method and purification procedure is more effective, quicker, and better, and it is especially applicable when the purification of target proteins is impractical or when the target protein concentration is low.

**Figure 4:**
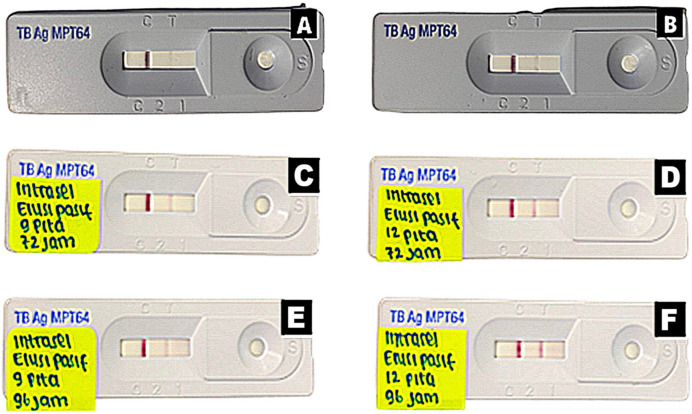
MPT64 protein immunochemical characterization intracellular electroelution results. (**A**) Electroelution with 9 bands; (**B**) electroelution with 12 bands; (**C**) passive elution with 9 bands for 72 h; (**D**) passive elution with 12 bands for 72 h; (**E**) passive elution with 9 bands for 96 h; (**F**) passive elution with 12 bands for 96 h.

The existence of anti-MPT64 antibodies that were detected using commercial MPT64 detection kits that are positively recognized serves as evidence of the MPT64 protein’s identification, as illustrated in Figure 4. This demonstrates that pure MPT64 protein can be produced using both elution techniques.

### 2.3. Detection of Linear MPT64 Epitope

The MPT64 protein purified using both approaches demonstrated a positive immunochemical response, demonstrating the insertion of certain antigens without altering the structure of the MPT64 protein epitope, as presented in Figure 4. These findings imply that IgG anti-MPT64 can recognize linear epitopes of the MPT64 protein. In addition, the isolated protein was confirmed as MPT64 protein based on the MALDI-TOF results. Neither of these two methods resulted in epitope changes in the MPT64 protein, so they could specifically react with both antibodies, and the amino acid sequences of its peptide were not affected by the denaturing purification treatment conditions (see Figure S1 in the Supplementary Materials). The stability of the MPT64 protein’s structure was also confirmed by the specific immunoassay result between pure MPT64 protein and IgY anti-MPT64.

**Figure 5:**
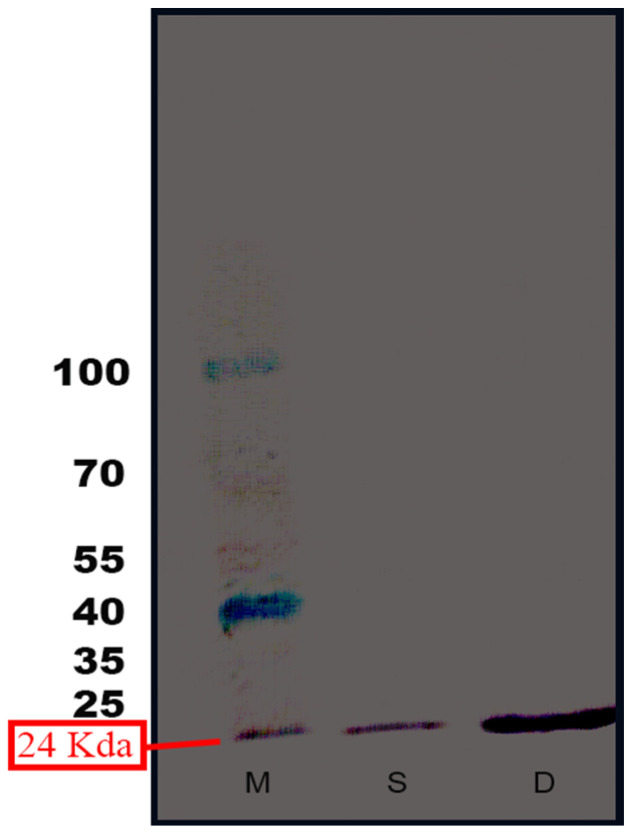
Western blot analysis: M = protein marker; S = pure isolated MPT64 protein; D = total protein extract.

This demonstrates that IgY anti-MPT64 can detect the isolated MPT64 protein as well as the MPT64 protein contained in the total protein extract from the source transformant without cross-reaction with other non-MPT64 proteins, as presented in Figure 5.

In both methods, the MPT64 protein is denatured into a linear monomer due to its contacted with anionic detergent sodium dodecyl sulfate (SDS) and polyacrylamide. Therefore, the structure of the antigen must also be considered because it affects the position of the epitope, which must be recognized by the antibody so that the antibody can react with the actual antigen structure in the clinical sample. The protein’s structure, and whether the linear or conformational epitope is recognized, has a significant impact on the performance and specificity of the antibody binding to its protein target [31]. In general, epitopes fall into one of two categories: conformational or linear. In conformational epitopes, important amino acid residues are brought together via protein folding, whereas in linear epitopes, a stretch of continuous amino acids is necessary for binding [32]. For applications requiring protein targets in their natural states, such as in therapeutic applications, conformational epitopes may be chosen. However, in situations where the protein target is completely or partially denatured during the sample preparation prior to the immuno test, linear epitopes may be preferable [31]. In particular, the diagnostic kits used to detect specific antigens as specific disease parameters are highly dependent on the immunochemical binding of specific antibody proteins to the target antigens. Antigens are typically used in immunochromatography for analyte detection because they can bind to IgG antibodies with specificity and can be strongly adsorbed onto nitrocellulose membranes. This attachment is accomplished via direct physical adsorption, allowing antibodies to be arbitrarily oriented in order to disrupt the active site of antibody binding. The success of MPT64’s binding to an antibody is significantly influenced by the stability of its structural conformation. Therefore, the development of all-inclusive purification and capture techniques is challenging due to the diversity of proteins and their biological characteristics. A good, precise, and reliable affinity ligand for capture onto a solid matrix is lacking for the majority of noteworthy proteins [33]. An antigen with correct folding might substantially react with excellent binding properties [34]. The location of the epitope on the MPT64 protein must be taken into consideration when analyzing purification data. Epitope alterations alter how antibodies are recognized and provide false-negative findings when antigen-based diagnostic kits are used. In this study, we determined that both extraction methods generate pure MPT64 protein with linear epitopes that are specifically recognized by the antibodies of anti-MPT64 proteins.

## 3. Conclusions

It can be inferred that the electroelution method is a more effective method of purifying MPT64 protein and maintaining its epitope to be precisely recognized by an antibody than the passive elution method. These methods are effective and easily obtain the purified tag-less protein. These methods can be used by experts in research groups and core facilities with substantial competence in (recombinant) protein purification to provide suitable starting points for obtaining pure, soluble proteins in amounts nearly in milligrams.

## 4. Materials and Methods

### 4.1. Materials

The materials used were Luria–Bertani broth and agar (LB broth and LB agar; Sigma -Aldrich, St. Louise, MO, USA), L-rhamnosa (Sigma-Aldrich, St. Louise, MO, USA), kanamycin sulfate (Sigma-Aldrich, St. Louise, USA), a dialysis membrane (Sigma-Aldrich, St. Louise, MO, USA), ethylenediaminetetraacetic acid (EDTA; Sigma-Aldrich, St. Louise, MO, USA), sodium bicarbonate (Sigma-Aldrich, St. Louise, MO, USA), and phosphate-buffered saline (PBS; Sigma-Aldrich, St. Louise, MO, USA).

### 4.2. Protein Overproduction

A starter culture was prepared by inoculating an *E. coli* BL21 (DE3) transformant [pD861-SR: 319895] in 10 mL of LB liquid, to which kanamycin was added (the kanamycin stock was 50,000 ppm) with a final concentration of 100 ppm. The culture was then incubated at 37 °C and shaken at 180 rpm for 5–7 h. The incubated cultures were observed for turbidity, and an OD600 measurement of 0.696 was obtained. To create a growth curve, the OD600 was examined for 0 to 8 h. Furthermore, 4 mL of the starter culture was inoculated into 1 L of 2× LB liquid medium containing 400 µL of kanamycin. The culture was incubated at 37 °C and shaken at 180 rpm. In the exponential phase, the OD600 was examined by sampling 1 mL of the culture. Then, rhamnose was added to the culture at the 5th h (the optimum induction time) to reach a final concentration of 4 mM. Then, re-incubation was carried out at 37 °C and with 180 rpm shaking. After 24 h, the culture turbidity was examined with an OD600 measurement. Then, cells were harvested by transferring the culture into a centrifuge tube.

### 4.3. Isolation of Intracellular MPT64 Protein

The obtained sample was put into a 50 mL centrifuge tube and then centrifuged for 20 min at a speed of 6000× *g* at 4 °C to separate the pellet and supernatant. The cell pellet was resuspended by adding 0.1 M Tris-Cl EDTA and 1 µL of PMSF lysis buffer to achieve a final concentration of 1 mM, sonicated for 15 min (15 s on, 15 s off), and then centrifuged at a higher speed 10,000× *g* at 4 °C for 30 min. The obtained supernatant was then separated and marked as the cytoplasmic fraction (FS). The FS was stored at −20 °C for further analysis. The MPT64 protein in the obtained total intracellular protein was characterized using a commercial MPT64 detection diagnostic kit and the SDS-PAGE method.

### 4.4. SDS-PAGE

SDS-PAGE was used to isolate the MPT64 protein from the crude intracellular protein extract into a single protein band. A separating gel (12%) and a stacking gel (4%) comprised the electrophoresis gel. The protein was diluted in 5 µL of a 5× buffer sample before being inserted into the hole, and it was then heated for 15 min at 95 °C. The reservoir was supplied with a 10 µL volume of the protein sample and the marker. At 100 V and 400 A, electrophoresis was performed for 90 min.

### 4.5. Passive Elution

Passive elution was carried out at 4 °C with different incubation times of 72 and 96 h to optimize the protein’s release into the elution buffer solution. Variable amounts, namely 6, 9, and 12 bands, of the MPT64 protein gel at 24 kDa were needed for each condition during incubation. The gel was sliced with a sterile knife and incubated in 100 μL (6 bands), 150 μL (9 bands), and 200 μL (12 bands) of sterile PBS solution. The gel debris was then centrifuged at 3000 rpm for 5 min to isolate the supernatant. After the incubation time was complete, the supernatant was separated from the gel debris and transferred into a sterile microtube, which was then stored in a freezer. The passively eluted MPT64 protein that was obtained was characterized using the SDS-PAGE method and a commercial MPT64 detection diagnostic kit. Protein content was measured using the nanodrop method.

### 4.6. Electroelution

The SDS-PAGE resulting gel was first cut with variations in the number of bands (6, 9, and 12) inserted into the activated dialysis membrane. Before use, the 10 cm membrane needed to be activated by adding 50 mL of 1 mM EDTA at pH 8 and 50 mL of 2% sodium bicarbonate to the membrane and then heating it at 60 °C for 10 min. The heated membrane was rinsed using distilled water, 100 mL of 1 mM EDTA was added, and then it was heated at 60 °C. The membrane was re-washed using distilled water. At this stage, the membrane was activated and could be used for electroelution. Then, the membrane dialysis tube was filled with 150 μL (9 bands) and 200 μL (12 bands) of sterile PBS solution, and the gel slices were inserted into the tube. The flap was tied on both sides using sewing thread. The closed membrane was placed in an electroelution apparatus containing an elution buffer. Electroelution was carried out for 3 h at room temperature and a 50 V voltage. When the electroelution process was completed, the membrane was opened, and the solution containing protein was stored in a sterile microtube to calculate the protein content. The electroelution results were characterized using the SDS-PAGE method and protein content measurements using the nanodrop method.

### 4.7. MPT64 Protein Detection Confirmation

The immunochromatographic kit SD Bioline TB Ag MPT64 was used to validate the presence of MPT64 protein in the cytoplasmic fraction in addition to the SDS-PAGE findings (Standard Diagnostics, Inc., Suwon, Republic of Korea). After the MPT64 protein in the SDS-PAGE gel band was separated, the extraction buffer from the kit was used to suspend it, and 100 L of the suspension was poured into the sample area. After 15 min, the generated colors of the bands on the control (C) and test (T) lines were assessed. The presence of two bands on the test and control lines indicated that the sample produced a positive result. As supporting data, the isolated protein was also identified with peptide mass fingerprinting using MALDI-TOF MS and reacted with IgG anti-MPT64 obtained from the commercial diagnostic kit, and the cross-reactivity of the isolated protein with IgY anti-MPT64 was confirmed using Western blot. IgY chicken anti-MPT64 was used as the primer antibody, and IgY rabbit alkaline phosphatase (Sigma-Aldrich) with alkaline phosphatase buffer, NBT, and BCIP were used as the substrates for WB visualization.

## Figures and Tables

**Table 1:** Intracellular MPT64 protein contents.

No.	Methods and Conditions	Content (µg/mL)		Content Average (µg/mL)
		1	2	
1	Total protein extract	25,405	25,425	25,415 ± 10
2	Electroelution with 12 bands	2071	1989	2030 ± 41
3	Electroelution with 9 bands	1151	1151	1151 ± 0
4	Passive elution with 12 bands, 96 h	172	187	179.5 ± 7.5
5	Passive elution with 12 bands, 72 h	138	119	128.5 ± 9.5
6	Passive elution with 9 bands, 96 h	111	93	102 ± 9
7	Passive elution with 9 bands, 72 h	96	93	94.5 ± 1.5
8	Passive elution with 6 bands, 96 h	58	58	58 ± 0
9	Passive elution with 6 bands, 72 h	46	57	51.5 ± 5.5

## Data Availability

Not applicable.

## Supplementary Materials

The following supporting information can be downloaded at: https://www.mdpi.com/article/10.3390/gels9070578/s1, Figure S1: MPT64 Blast.
