# Supplementary material for: Gel Protein Extraction’s Impact on Conformational Epitopes of Linear Non-Tagged MPT64 Protein
Source: Gels. 2023 Jul 14;9(7):578. doi: 10.3390/gels9070578 (PMC10378983; doi:10.3390/gels9070578)
Supplement: Supplementary file 1 [file gels-09-00578-s001.zip › Figure S1.pdf]

BLAST ® » **blastp suite** » results for RID-70FMXFRJ013

Your search parameters were adjusted to search for a short input sequence.
Your results are filtered to match records that include: Mycobacterium tuberculosis H37Rv (taxid:83332)

**Job Title**	mpt64 malditof ...
**RID**	70FMXFRJ013 Search expires on 05-27 13:07 pm
**Program**	BLASTP
**Database**	nr
**Query ID**	lcl|Query_72317
**Description**	unnamed protein product ...
**Molecule type**	amino acid
**Query Length**	16

Compare these results against the new Clustered nr database BLAST

Descriptions

Description	Scientific Name	Max Score	Total Score	Query Cover	E value	Per. Ident	Acc. Len	Accession
immunoprotective protein Mpt64 [Mycobacterium tuberculosis complex]	Mycobacterium tuberculosis complex	57.1	57.1	100%	7e-08	100.00%	228	**WP_003409954.1**

Graphic Summary

Distribution of the top 1 Blast Hits on 1 subject sequences

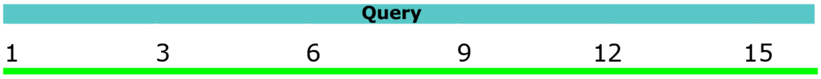

Alignments

Alignment view Pairwise ☐ CDS feature Restore defaults

MULTISPECIES: immunoprotective protein Mpt64 [Mycobacterium tuberculosis complex]
Sequence ID: **WP_003409954.1** Length: 228 Number of Matches: 1
Range 1: 1 to 16

Score	Expect	Identities	Positives	Gaps	Frame
57.1 bits(127)	7e-08()	16/16(100%)	16/16(100%)	0/16(0%)	

```
Query  1    MRIKIFMLVTAVVLLC  16
            MRIKIFMLVTAVVLLC
Sbjct  1    MRIKIFMLVTAVVLLC  16
```

Taxonomy

Reports

- **Lineage**

Organism	Blast Name	Score	Number of Hits	Description
Mycobacterium	high G+C Gram-positive bacteria		3	
.Mycobacterium tuberculosis complex	high G+C Gram-positive bacteria	57.1	1	**Mycobacterium tuberculosis complex hits**
.Mycobacterium tuberculosis H37Rv	high G+C Gram-positive bacteria	57.1	2	**Mycobacterium tuberculosis H37Rv hits**

- ## Organism

Description	Score	E value	Accession
Mycobacterium tuberculosis complex [high G+C Gram-positive bacteria]			
**MULTISPECIES: immunoprotective protein Mpt64 [Mycobacterium tuberculosis complex]**	57.1	7e-08	**WP_003409954**
Mycobacterium tuberculosis H37Rv [high G+C Gram-positive bacteria]			
**immunogenic protein Mpt64 [Mycobacterium tuberculosis H37Rv]**	57.1	7e-08	**NP_216496**
**RecName: Full=Immunogenic protein MPT64; AltName: Full=Antigen MPT64; Flags: Precursor [Mycobacterium tuberculosis H37Rv]**	57.1	7e-08	**P9WIN9**

- ## Taxonomy

Taxonomy	Number of hits	Number of Organisms	Description
Mycobacterium	3	2	
. Mycobacterium tuberculosis complex	1	2	**Mycobacterium tuberculosis complex hits**
.. Mycobacterium tuberculosis H37Rv	2	1	**Mycobacterium tuberculosis H37Rv hits**
